# A molecular characterization of marsupial filarioid nematodes of the genus *Breinlia*

**DOI:** 10.1017/S0031182022001482

**Published:** 2023-01

**Authors:** Anson V. Koehler, Ian Beveridge, David M. Spratt

**Affiliations:** 1 Department of Veterinary Biosciences, School of Veterinary Science, Faculty of Veterinary and Agricultural Sciences, The University of Melbourne, Victoria, Australia; 2 Australian National Wildlife Collection, Commonwealth Scientific and Industrial Research Organisation, Canberra, Australia

**Keywords:** Australia, filarioidea, marsupial, nematode, phylogeny

## Abstract

Here we present the genetic relationships of 26 specimens of the genus *Breinlia* (Nematoda: Filarioidea) from a range of Australian marsupials using markers in the small subunit of nuclear ribosomal RNA and mitochondrial cytochrome *c* oxidase subunit 1 (*cox*1) genes and compare them with morphological determinations. The molecular data support the validity of most of the morpho-species included in the study and provide provisional insights into the phylogeny of the genus in Australian mammals, with dasyuroid marsupials appearing to be the original hosts. The recent discovery of *Breinlia annulipapillata* in the eye of a human brings this genus of parasites into the group of emerging infectious parasitic diseases.

## Graphical abstract

**Figure:**
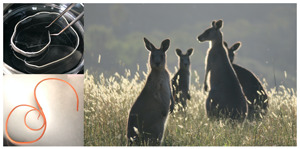

## Introduction

Filarioids are a highly diverse group of parasitic nematodes found primarily in the interstitial tissues and vascular system of terrestrial vertebrates (Bain *et al*., 2013), with the group being particularly well represented in Australasian mammals (Spratt and Varughese, 1975; Spratt, 2011). At the time of the first reviews of the Australian filarioid fauna (Mackerras, 1962; Spratt and Varughese, 1975), most species were accommodated within the large genus *Dipetalonema*. Comparing morphological evolution with host range and geographic distribution of the genus *Dipetalonema sensu lato*, Chabaud and Bain (1976) suggested that it was a Gondwanan lineage which probably diversified after the 3 austral continents drifted apart. On this basis, they undertook a major taxonomic revision of the genus which included the recognition of *Breinlia* (*Breinlia*) and *Breinlia* (*Johnstonema*), both present in Australasian mammals but previously included within *Dipetalonema*. Their treatment of these groups was based on earlier work on the Australian species of the genus (Spratt and Varughese, 1975). To date, 22 species of *Breinlia* (*Breinlia*) are known from marsupial and murid hosts in Australia and Papua New Guinea (Spratt, 2011), 5 from murid and sciurid hosts in Southeast Asia, and 2 from lorisid and sciurid hosts in India (Veciana *et al*., 2015). Two species of *Breinlia* (*Johnstonema*) have been described from macropodid marsupials in Australia and 2 unnamed species, 1 from Papua New Guinea, are recognized (Spratt, 2011).

Among the Australasian marsupials, *Breinlia* (*Breinlia*) achieves its greatest diversity in kangaroos and wallabies (Macropodidae) with 14 species reported (Spratt, 2011). Apart from the macropodid hosts, 2 species are known from dasyurids, 1 from bandicoots (Peramelidae), 1 each from petaurids, phalangerids and pseudocheirids (possums) and 4 from potoroids (potoroos) (Spratt, 2011). Additional un-named species are also known (Spratt, 2011). A number of filarioid occurrences classified as accidental have been recognized in koalas (Phascolarctidae) (Spratt, 2011) and no filarioids are known from wombats (Vombatidae), both families being members of the sub-order Vombatiformes.

The subgenera are distinguished on morphological features, particularly those of the male. In *Breinlia* (*Breinlia*) the spicules are unequal, not stout, the left spicule is divided into calomus, lamina and filament, the right spicule has a spatulate distal extremity and a gubernaculum is present. In *Breinlia* (*Johnstonema*), the spicules are subequal, stout and not divided into calomus, lamina and filament, the right spicule is without a spatulate distal extremity and a gubernaculum is absent (Spratt, 2011).

While the subgenera are readily distinguished on morphological features, species differentiation can be more difficult, especially in *Breinlia* (*Breinlia*). Here, body length and features of the cephalic end viewed *en face* are useful in both sexes. The most reliable characteristics have been the length of the left and right spicules, the ratio of one to the other and the relative lengths of the 3 components of the left spicule (Spratt and Varughese, 1975). However, differentiation between some species pairs (*B. boltoni*, *B. mundayi*) can be difficult based on morphological characters (Spratt, 2011).

Molecular data would be valuable, not only in defining species limits within *Breinlia*, but also in providing insight into the phylogenetic origins of the genus, particularly the diversification within the kangaroos and wallabies. However, DNA sequence data are currently available for only 4 species of *Breinlia*: *B. mundayi* (in Laetsch *et al*., 2012), *B. jittapalapongi* (Lefoulon *et al*., 2015), *B. annulipapillata* (in Koehler *et al*., 2021) and *Breinlia* sp. (Steventon *et al*., 2021). The recent finding of *B. annulipapillata* in the eye of a human brings this genus of nematode parasites to the category of emerging infectious diseases (Koehler *et al*., 2021). Here, we present the phylogenetic relationships of 12 morphospecies of *Breinlia* spp. based on the small subunit of the nuclear ribosomal RNA gene (*SSU*) and mitochondrial cytochrome *c* oxidase 1 gene (*cox*1) and compare them with morphological determinations.

## Materials and methods

### Specimen collection

Nematodes were collected from commercially killed and road killed marsupials between 1989 and 2018. Specimens were collected under the following permit numbers: Queensland: National Parks and Wildlife Service T-00436, T-00759, T-00943, T-1131, Department of Environment and Science WA0006125; Victoria: Department of Environment and Conservation, Department of Sustainability and Environment, Department of Environment, Land, Water and Planning RP-92-018, RP-93-016, RP-95-039, RP-97-046, 10008033; Northern Territory: Parks and Wildlife Commission 15747; Western Australia: Department of the Environment and Conservation SF007407; South Australia: National Parks and Wildlife Service E07358.

Voucher specimens were cleared in lactophenol and examined morphologically and have been deposited in the National Wildlife Collection, CSIRO, Canberra (N5508, 5694, 5745, 5764-5771). Morphological identifications followed Spratt and Varughese (1975) and Spratt (2011). Host nomenclature follows Jackson and Groves (2015).

### DNA extraction

DNA was extracted from both frozen and ethanol-fixed specimens of *Breinlia*. Ethanol-fixed specimens were washed 3 times in double-distilled water (ddH_2_O) prior to extraction. Worms were placed in 400 *μ*L of extraction buffer [20 mm Tris–HCl (pH 8.0), 100 mm EDTA and 1% SDS] and 20 *μ*L of proteinase K 20 mg mL^−1^ MC5005 (Promega, Fitchburg, Wisconsin, USA) overnight with several vortex steps. The extraction proceeded as per the manufacturer's instructions in the Promega Wizard DNA clean-up kit (A7280). The final elution step was performed with 50 *μ*L of ddH_2_O and then repeated through the same column to increase DNA yield.

### Polymerase chain reaction and sequencing

Selected *Breinlia* specimens were characterized using a partial region of *SSU* (cf. Lefoulon *et al*., 2015) employing primers F18ScF1 (forward: 5′-ACC GCC CTA GTT CTG ACC GTA AA-′3) and F18ScR1 (reverse: 5′-GGT TCA AGC CAC TGC GAT TAA AGC-′3) using the following cycling protocol: 94°C for 5 min (initial denaturation), followed by 35 cycles of 94°C for 30 s (denaturation), 58°C for 45 s (annealing) and 72°C for 1 min (extension), with a final extension of 72°C for 5 min. All samples were characterized with a nested polymerase chain reaction (PCR) assay targeting 650 bp of *cox*1 employing primers FCo1extdF1 (forward: 5′-TAT AAT TCT GTT YTD ACT A-′3) and FCo1extdR1 (reverse: 5′-ATG AAA ATG AGC YAC WAC ATA A-′3) in the primary PCR (Lefoulon *et al*., 2015) and primers COIintF (forward: 5′-TGA TTG GTG GTT TTG GTA A-′3) and COIintR (reverse: 5′-ATA AGT ACG AGT ATC AAT ATC-′3) in the second PCR (Casiraghi *et al*., 2001). Both PCRs employed the following cycling protocol: 94°C for 5 min (initial denaturation), followed by 35 cycles of 94°C for 30 s (denaturation), 52°C for 45 s (annealing) and 72°C for 1 min (extension), with a final extension of 72°C for 5 min. All PCRs were conducted in a volume of 50 *μ*L containing 2 *μ*L of DNA, GoTaq Flexi buffer (Promega), 3.0 mm of MgCl_2_, 200 *μ*m of each deoxynucleotide triphosphate, 25 pmol of each primer and 1 U of GoTaq DNA polymerase (Promega). Known test-positive (*Breinlia* sp. DNA), test-negative and no-template controls were included in each PCR run. The intensity and size of all amplicons were assessed by agarose electrophoresis. PCR products were sequenced bi-directionally using a standard protocol (Koehler *et al*., 2016).

### Assembly and tree construction

Sequences were preliminarily assessed by comparing them to publicly available sequences from the GenBank database at the National Center for Biotechnology Information (NCBI). The *cox*1 (652 bp) sequences obtained were separately aligned with reference sequences representing distinct filarioid species and *Mansonella ozzardi* as the outgroup obtained from the NCBI database. Sequences were aligned using Muscle (Edgar, 2004), and adjusted manually within the program Mesquite v.3.61 (Maddison and Maddison, 2015). Phylogenetic analyses of sequence data were conducted by Bayesian inference (BI) using Monte Carlo Markov Chain analysis in the program MrBayes v.3.2.6 (Ronquist *et al*., 2012). The likelihood parameters set for BI analyses of sequence data were based on the Akaike information criteria test in IQ-TREE v.2 (Minh *et al*., 2020), with the number of substitutions (Nst) set at 6 and a *γ* distribution. Posterior probability (pp) values were calculated by running 2 000 000 generations with 4 simultaneous tree-building chains. Trees were saved every 100th generation. At the end of each run, the standard deviation of split frequencies was <0.01, and the potential scale reduction factor approached 1. For each analysis, a 50% majority rule consensus tree was constructed based on the final 75% of trees generated by BI. Analyses were run 3 times to ensure convergence and insensitivity to priors. Pairwise differences generated with Geneious Prime v2022.1.1 can be found in Supplementary Table S1.

## Results

### Genetic characterization using *SSU* sequences

Most of the *SSU* sequences (ranging in lengths from 660 to 718 bp) were identical to that of *Breinlia* sp. isolate LBF5 from the Leadbeater's possum (GenBank accession no. MT731343) (Steventon *et al*., 2021). Sequence 6Y4, *Breinlia boltoni* from *Notamacropus agilis*, matched *Breinlia mundayi* with 100% identity (GenBank accession no. JF934735). Selected sequences were deposited in GenBank under accession nos. OP069989–OP069999.

### Phylogenetic relationships using *cox*1 sequence data

**Fig. 1.**
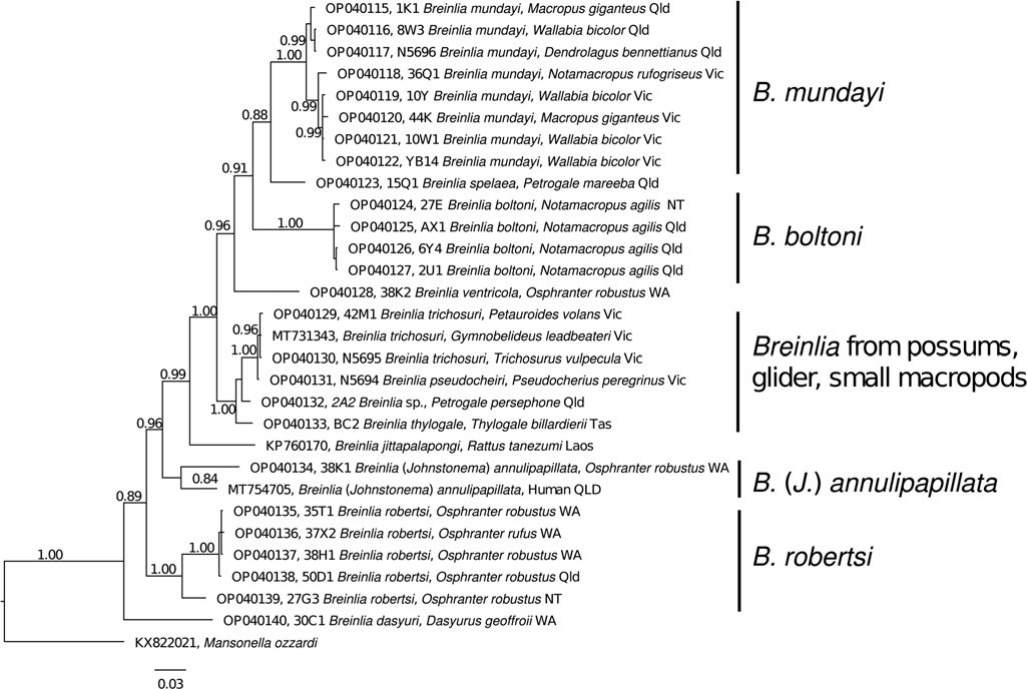
Phylogenetic relationships of members of the *Breinlia* genus from a range of marsupial hosts collected throughout Australia, along with representative sequences from the GenBank database, based on an analysis of a partial region of the cytochrome *c* oxidase 1 gene (*cox*1) employing the Bayesian method. Branch supports are Bayesian posterior probabilities. The closely related filarioid nematode, *Mansonella ozzardi* was used as the outgroup. GenBank accession number is followed by field number.

Twenty-six novel (GenBank accession nos. OP040115–OP040140) and 3 existing (GenBank nos. MT731343, MT754705 and KP760170) partial *cox*1 sequences were aligned with *M. ozzardi* as the outgroup (GenBank no. KX822021). Sequences were 652 bp in length except for *B. robertsi* (GenBank no. OP040135; 651 bp), *B. trichosuri* (GenBank no. OP040130; 633 bp) and *B*. *jittapalapongi* (GenBank no. KP760170; 596 bp). The resulting *cox*1 phylogenetic tree can be divided into 5 strongly supported clades [*B. mundayi* clade, *B. boltoni* clade, the possum/glider/rock-wallaby/pademelon clade, *B*. (*J*.) *annulipapillata* clade and *B. robertsi* clade] with individual sequences interspersed amongst them (*B. spelaea*, *B. ventricola*, *B. jittapalapongi* and *B. dasyuri*) (Fig. 1). The *B. mundayi* clade is comprised of 8 sequences which can be further subdivided geographically between 3 sequences from Queensland and 5 from Victoria. *Breinlia spelaea* falls between the *B. mundayi* clade and the *B. boltoni* clade. The *B. boltoni* clade consists of 4 samples, all from *Macropus agilis* collected from Queensland and the Northern Territory. *Breinlia ventricola* lies between the *B. boltoni* clade and the possum/glider/rock-wallaby/pademelon clade. *Breinlia thylogale*, from a Tasmanian pademelon *Thylogale billardierii*, is most closely related to *Breinlia* sp. from the Proserpine rock-wallaby, *Petrogale persephone* which together are distinct from the 4 possum and glider sequences including *B. trichosuri* and *B. pseudocheiri*. There is little (2 bp) to no difference amongst the 4 glider and possum sequences (Supplementary Table S1). *Breinlia jittapalapongi* from *Rattus tanezumi* from Laos sits between this clade and the *B*. (*J*.) *annulipapillata* clade consisting of 2 distantly related sequences (93.1% similarity with 45 bp differences: Supplementary Table S1), one from a human in Queensland and the other from *Osphranter robustus* in Western Australia. The *B. robertsi* clade consists of 4 closely related sequences and 1 moderately different sequence (95% identity with 32–34 bp differences from *O. robustus* of the Northern Territory; Supplementary Table S1). The most early divergent sequence in this tree is a lone *B. dasyuri* from a western quoll (*Dasyurus geoffroii*) from Western Australia.

## Discussion

The data presented here represent the first investigation into the relationships of some of the Australian species of *Breinlia* using molecular methods. Although a limited range of species from marsupials was available, and none from murid rodents, the data provide some insights into both species' boundaries and phylogenetic relationships. As no meaningful differences were noted in the *SSU* sequences between samples (matching to either *Breinlia* sp. isolate LBF5 or *B. mundayi*), the analysis was based entirely on the *cox*1 gene. Of note from the present study was that it was possible to recover DNA relatively consistently from filarioid nematodes which had been fixed in ethanol and stored at room temperature for more than 20 years.

### Species boundaries

In most instances, the molecular data supported the validity of the species currently defined by morphological data. The validity of *Breinlia annulipapillata*, *B. boltoni*, *B. dasyuri*, *B. robertsi*, *B. spelaea*, *B. thylogale* and *B. ventricola* is each supported by the molecular data provided here. The data also suggest that the specimens identified to date as *Breinlia* sp. from *Petauroides volans* and *Gymnobelideus leadbeateri* are referrable to *B. trichosuri*, although adult males were not present to confirm this allocation. In the case of *P. volans* only female nematodes were available and in the case of *G. leadbeateri*, the identification was based on DNA sequence data from microfilariae (Steventon *et al*., 2021). *Breinlia pseudocheiri* was the sister taxon to the specimens of *B. trichosuri* in the phylogenetic analysis with high nodal support (1.00, Fig. 1) and the 2 genetically closely related species, differing by 2 base pairs only, formed a small sub-clade from possums and gliders (*Gymnobelideus*, *Petauroides*, *Pseudocheirus* and *Trichosurus*). However, although *B. trichosuri* and *B. pseudocheiri* are similar in terms of general morphology, the former is a much larger species in most of its morphological measurements and they also differ in their cephalic structures seen in *en face* preparations (Spratt and Varughese, 1975; Spratt, 2011).

*Breinlia pseudocheiri* has been recorded from the peritoneal and pleural cavities of most sub-species of ringtail possums, *Pseudocheirus peregrinus* from all states but not from hosts in the Australian Capital Territory or the Northern Territory (Spratt, 2011). The nematode has a wide geographic distribution among pseudocheirid and petaurid hosts, especially in coastal, southeastern Australia and is known from *P. volans* in Queensland, New South Wales and Victoria, which contrasts with the identification of the current specimens from this host species as *B. trichosuri*. *Breinlia pseudocheiri* exhibits a great deal of variation in measurements within a host species, between host subspecies and between host species (Spratt, 2011) although this variation does not encompass *B. trichosuri*. The host reaction surrounding some nematodes recovered from *P. peregrinus*, something not observed in *P. volans*, prompted Spratt (2011) to suggest that the latter may be the normal host. As only single specimens of *B. pseudocheiri* and *B. trichosuri* were available and only a single genetic region was examined, additional material of these 2 species is required to establish their apparent very close genetic similarity in spite of being morphologically distinct.

*Breinlia thylogale* from *T. billardierii* and *Breinlia* sp. from *P. persephone* were placed as sister species to the *B. pseudocheiri*/*B. trichosuri* clade. *Breinlia pseudocheiri* and *B. thylogale* are similar morphologically but are distinguished by the shorter left spicule with shorter filament, longer right spicule and longer microfilaria in *B. pseudocheiri*. *Breinlia thylogale* is known to occur in 2 species of pademelons, *T. billardierii* in Tasmania and *T. stigmatica* in Queensland (Spratt, 2011). However, only material from *T. billardierii* was available for the current study.

Specimens of *Breinlia* from *P. persephone*, originally identified by Spratt (2011) as *B. spelaea*, differed genetically from specimens of the same putative species from the other rock wallaby host, *P. mareeba*, included in this study. The same nematode species, *B. spelaea*, has also been reported from additional members of the ‘penicillata’ group of rock wallabies, *P. assimilis*, *P. herberti*, *P. inornata*, *P. pearsoni* and *P. sharmani*, occurring along the eastern coast of Australia (Spratt and Beveridge, 2016). However, molecular studies of several species of *Cloacina* from these same rock wallaby hosts have suggested that they each constitute a species complex (Chilton *et al*., 2009) with a different nematode species in each host species. Given the phylogenetic differences between *P. persephone* and its congeners (Eldridge and Close, 1997), its unusual habitat in rain and vine forests (van Dyck and Strahan, 2008), as well as the published molecular study on cloacinine nematodes of these same hosts, the affinities within this taxon warrant further investigation.

Spratt (2011) reported that *B. boltoni* was very similar morphologically to *B. mundayi*, the former predominantly a parasite of northern macropodoid species and the latter of southern members of the Macropodidae. The only specimens of *B. mundayi* from Queensland known at the time were from the swamp wallaby, *Wallabia bicolor*, the most common southern host of this nematode species (Spratt, 2011) and one in which *B. boltoni* had not been observed. The current data suggest that while the representatives of *B. mundayi* from southern Australia are distinct from *B. boltoni* in northern Australia, the current identifications of *B. mundayi* from northern Queensland may represent a distinct species. *Breinlia boltoni* was examined from 3 neighbouring localities near Townsville and Ingham in Queensland and 1 from the Northern Territory in the current study, but little molecular variation occurred within the clade including these specimens.

The validity of *B. ventricola* was also supported by the molecular data. *Breinlia ventricola* resembles *B. trichosuri*, *B. mundayi* and *B. boltoni* morphologically, but is distinguished from all 3 by its much greater size and absence of a pair of internolateral cephalic papillae. It is the most characteristic species of the subgenus due to the very large size of both males and females, the presence of 2 large caudal glands in both sexes and its occurrence in the right ventricle and pulmonary arteries of *O. robustus* and red kangaroo (*Osphranter rufus*) in Western Australia (Spratt and Hobbs, 2004; Spratt, 2011).

The molecular data suggest that specimens of *Breinlia* from *O. robustus* and *O. rufus* from Western Australia and Queensland, for which only female specimens were available, are identifiable as *B. robertsi*. However, the material of *B. robertsi* from *O. robustus* from the Northern Territory was genetically distinct from that collected in Queensland and Western Australia and the differences therefore warrant further investigation. The material from the Northern Territory was from a distinct sub-species of *O. robustus*, *O. r. woodwardi*, while that from Western Australia was from *O. r. erubescens* and the Queensland material was from *O. r. robustus*. Additional collections are required to examine the status of the material from the Northern Territory.

### Phylogeny

The phylogenetic analysis resulted in 5 well-supported clades (Fig. 1) with *B*. *dasyuri* from the quoll, *D. geofroii*, as sister to all the remaining species, while the species from possums and gliders (*B*. *pseudocheiri* and *B*. *trichosuri*) formed a sub-clade nested within the various clades from macropodoids and an Asian rodent.

Although the subgenera *Breinlia* (*Breinlia*) and *Breinlia* (*Johnstonema*) can be clearly defined morphologically (Spratt, 2011), this distinction is not evident in the molecular data which placed *B*. (*Johnstonema*) nested within a series of clades containing species of *B*. (*Breinlia*).

*Breinlia ventricola*, the sole cardiac-inhabiting species included in the study, occurred between the *B. boltoni* clade and the possum–macropodid clade in the phylogenetic analysis possibly related to its cardiac localization and the distinctive morphological features described above. Representatives of *B. annulipapillata* formed a separate clade and again, their sub-cutaneous localization and distinctive spicule morphology may be related to this phylogenetic position. Although there appears to be no clear relationship in the molecular phylogeny with localization within the host, most of the species included in the study are parasites of the abdominal and thoracic cavities. The occurrence of *B. ventricola* in the heart and pulmonary artery and the sub-cutaneous localization of *B. annulipapillata* may be reflected by their occurrence in distinctive branches within the molecular analysis.

The clade including the nematodes from possums and gliders (*B. pseudocheiri* and *B. trichosuri*) as well as *B. spelaea* from a rock wallaby and *B. thylogale* from the Tasmanian pademelon, *T. billardierii*, included a variety of host families (Pseudocheiridae, Phalangeridae, Macropodidae) and in this respect was unusual as most other major clades included hosts from single families or genera, apart from the occurrence of *B. anulipapillata* in a human.

Although taxon sampling was limited, the finding that *B. dasyuri*, from a dasyurid marsupial, is sister to all of the remaining taxa, suggests a possible phylogenetic origin for the Australasian representatives in dasyurids (Dasyuromorphia) but additional species of *Breinlia* from dasyurids would need to be added to confirm this suggestion. This hypothesis would be concordant with the views of Chabaud and Bain (1976) and Bain *et al*. (1982), who considered *Breinlia* to be of Gondwanan origin, and the phylogeny of the hosts, with the dasyuroids occurring earlier in the fossil record than the diprotodont marsupials (Vombatiformes, Phalangerida and Macropodiformes), the hosts of the remaining species included in this study (Beck, 2008) (apart from 1 species from an Asian rodent). No representatives of *Breinlia* spp. from bandicoots (Peramelimorphia) (*B*. *mackerrasae*) were available for the present study. The bandicoots predate the diprotodonts but not the dasyuroids in the fossil record (Beck, 2008) and harbour a diversity of other genera of filarioid nematodes apart from the single species of *Breinlia* (*B.*) *mackerrasae* (Spratt, 2011).

No species of *Breinlia* are known primarily from the Vombatiformes, the wombats (*Vombatus* and *Lasiorhinus*) and the koala (*Phascolarctos*) (with the exception of a single record of *B. mundayi* from the koala, thought to be an unusual host) (Spratt and Beveridge, 2016). The major radiation of the Australian species of *Breinlia* therefore appears to have been within the Macropodiformes (kangaroos, wallabies and rat-kangaroos) with a secondary invasion of the Phalangerida (possums and gliders) which predate the Macropodiformes within the fossil record (Beck, 2008), making a co-evolutionary hypothesis highly implausible. A similar pattern is also exhibited by the herpetostrongylid nematodes based on currently available morphological phylogenies (Beveridge and Durette-Desset, 1986).

The inclusion of a single species from an Asian rodent (*B. jittapalapongi*) also appears to be a secondary invasion of rodents, although data from species found in Australian rodents have yet to be added. Three species of *Breinlia* occur in Australian rodents (*B. melomyos*, *B. presidentei* and *B. zyzomyos*) but none was available for study. Rodents arrived in Australia relatively recently from the Sahal region to the north of the continent, probably only 5 million years ago (Rowe *et al*., 2008) and their acquisition of species of *Breinlia* may be an even more recent phenomenon, based on the current data from a single species of *Breinlia* from an Asian rodent. The species of *Breinlia* occurring outside the Australian region include 6 species from murid and sciurid rodents in south-east Asia (*B. booliati*, *B. jittapalapongi*, *B. manningi*, *B. petauristi, B. spratti*, *B. tinjili*) and *B. sergenti* from a loris (Lorisidae) from India (Veciana *et al*., 2015). Their association with the 22 species known from Australian mammals remains to be determined.

An obvious limitation to the current initial molecular study of Australian species of *Breinlia* is the limited taxon coverage as well as the reliance on a single mitochondrial gene for inferring phylogenetic relationships. In addition, the lack of data from species parasitic in Australian bandicoots and rodents represents a particular deficiency. However, the data available do suggest a possible origin in dasyuroid marsupials and hence a long association with the Australian marsupial fauna.

## Data Availability

Sequences used in this study are available *via* GenBank accession numbers OP040115–OP040140 (*cox*1) and OP069989–OP069999 (*SSU*).

## Supplementary material

For supplementary material accompanying this paper visit https://doi.org/10.1017/S0031182022001482. click here to view supplementary material
